# Unnecessary magnetic resonance imaging is associated with diagnostic delay and financial burden in benign paroxysmal positional vertigo: a retrospective study in Mongolia

**DOI:** 10.3389/fneur.2026.1777303

**Published:** 2026-02-13

**Authors:** Delgerzaya Enkhtaivan, Dong Woo Nam, Jargalkhuu Erdenechuluun, Tovuudorj Avirmed, Zaya Makhbal, Sainbileg Chadraabal, Baigal Minjuur, Tergel Nayanjin, Ja-Won Koo

**Affiliations:** 1 Department of Otorhinolaryngology, Mongolian National University of Medical Science, Ulaanbaatar, Mongolia; 2 Department of Otorhinolaryngology, EMJJ-ENT Hospital, Ulaanbaatar, Mongolia; 3 Department of Otorhinolaryngology-Head and Neck Surgery, Seoul National University Bundang Hospital, Seoul National University College of Medicine, Seoul, Republic of Korea; 4 Department of Otorhinolaryngology, Graduate School of Medicine, Chungbuk National University, Cheongju, Republic of Korea; 5 Department of Neurology, Mongolian National University of Medical Science, Ulaanbaatar, Mongolia

**Keywords:** benign paroxysmal positional vertigo, developing country, diagnostic delay, Dizziness Handicap Inventory, economic burden, Mongolia, videonystagmography

## Abstract

**Introduction:**

This single-center, retrospective cohort study investigated the clinical characteristics, diagnostic pathways, and economic burden of benign paroxysmal positional vertigo (BPPV) at a national tertiary referral ENT hospital in Ulaanbaatar, Mongolia.

**Methods:**

We analyzed 162 patients (mean age, 50.0 ± 11.7 years) with confirmed BPPV who completed follow-up between January 2019 and January 2021. Patients were treated with canalith repositioning procedures and instructed to perform self-maneuvers. The primary outcome was symptom resolution at 7, 14, and 28 days, and the secondary outcome was the change in Dizziness Handicap Inventory (DHI) scores.

**Results:**

Results showed that 102 patients (62.5%) were referred from other hospitals, yet only 6 (3.7%) were correctly diagnosed prior to referral. Multivariate logistic regression identified no specific clinical symptoms predicting magnetic resonance imaging (MRI) usage, suggesting that neuroimaging was largely driven by patient-initiated demand in the private sector. The mean diagnostic delay was significantly longer in the MRI group (24.4 ± 19.5 days) compared to the non-MRI group (7.5 ± 5.9 days, *p* < 0.001). Cost analysis based on 2024 metrics revealed that a single potentially low-yield MRI consumes 72.0% of a minimum-wage worker’s monthly income, creating a catastrophic financial burden. Treatment was highly effective, with resolution rates of 75.9, 93.8, and 99.4% at 7, 14, and 28 days, respectively. The mean DHI score improved significantly from 39.93 to 4.12 (*p* < 0.001).

**Discussion:**

BPPV patients in Mongolia face significant diagnostic delays and high misdiagnosis rates. While standardized maneuvers are effective, the reliance on costly imaging highlights an urgent need for educational initiatives to improve awareness and primary care triage in developing countries.

## Introduction

A fundamental understanding of vestibular physiology and the clinical manifestations of various causes of dizziness is essential for the accurate evaluation of patients with this condition. When clinicians lack familiarity with dizziness evaluation and fail to recognize the nystagmus associated with benign paroxysmal positional vertigo (BPPV), affected patients may undergo unnecessary tests and treatments. Unnecessary diagnostic procedures have been reported in more than 65% of patients with BPPV, even in advanced countries (1, 2). One study showed that 70% of patients underwent magnetic resonance imaging (MRI), 45% underwent computed tomography, 41% underwent electrocardiography, and 53% received medication during the evaluation of BPPV (3). Improvements to the diagnosis and treatment of BPPV, the most common cause of dizziness, could reduce healthcare costs and enhance the quality of care. Other problems, such as anxiety, depression, and fall-related traumatic injuries, could be prevented or better managed. Affective symptoms, including depression, demoralization, phobia, anxiety, and somatization, are highly prevalent in patients with BPPV (4–6). BPPV also negatively affects sleep quality, psychological well-being, and the risk of falls. The consequences of BPPV include restricted daily activities, worsening disease prognosis, and an increased likelihood of falling (4, 7). Another study demonstrated that residual dizziness, a common condition of unknown origin, often presents as persistent disabling imbalance after successful repositioning maneuvers for BPPV. Decreased postural control impacts quality of life by contributing to falls and psychological problems (8, 9).

Although otolaryngologic care has been available in Mongolia for over 70 years (since 1950), there has been no vestibular specialist or laboratory facility to evaluate patients with dizziness for most of this period. Furthermore, the concept of vestibular physiology and knowledge of BPPV have not been widely disseminated. Historically, all patients with dizziness were generally treated in the same manner, primarily through the prescription of vestibular suppressants. This approach hindered the effective diagnosis, treatment, and monitoring of treatment outcomes; the duration of treatment was often prolonged because BPPV was managed solely based on general clinical symptoms.

The first vestibular laboratory in Mongolia was established in 2016 at the ENT Hospital, a specialized ear, nose, and throat hospital in Ulaanbaatar. Since then, peripheral vestibular diseases have been more effectively diagnosed and treated. This study reviewed the current status of medical services for BPPV in Mongolia, the most common and straightforward cause of dizziness, and explored the process of establishing a dizziness clinic in a developing country.

## Materials and methods

### Study design

This was a retrospective cohort study conducted at a single secondary referral center in Mongolia. This study was approved by the Research Ethical Committee of the MNUMS on 21 December 2018 (No. 2018/3-18). We retrospectively analyzed prospectively collected data from patients diagnosed with BPPV. Informed consent was obtained from all participants.

### Patients

Patients were recruited between January 2019 and January 2021 at ENT Hospital. The diagnosis of BPPV was made in accordance with the 2017 American Academy of Otolaryngology–Head and Neck Surgery Foundation (AAO–HNSF) BPPV guidelines. The inclusion criteria were age ≥18 years, agreement to participate in the study, and completion of follow-up evaluations.

**Figure 1:**
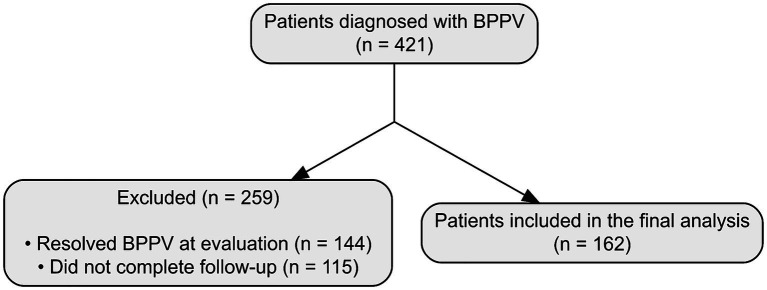
Flow diagram of patient enrollment. The diagram shows the patient selection process for the study. A total of 421 patients were initially diagnosed with benign paroxysmal positional vertigo (BPPV). Among them, 259 patients were excluded: 144 because their BPPV had already resolved at the time of evaluation, and 115 who were lost to follow-up. Consequently, 162 patients were included in the final analysis.

BPPV was diagnosed in 421 patients. Among these, 144 patients who did not exhibit positional vertigo or nystagmus at the time of evaluation (resolved BPPV) and 115 patients who did not complete follow-up examinations were excluded. Consequently, 162 patients were included in this study (Figure 1). Clinical questionnaires and the Dizziness Handicap Inventory (DHI) were completed, and videonystagmography was recorded and analyzed for all patients.

### Patient and public involvement

No patients or members of the public were involved in the design, conduct, reporting, or dissemination plans of this research.

### Clinical questionnaire

The questionnaire consisted of three parts. The first part collected general patient information, including name, age, sex, and address. The second part gathered details about the history of the current illness, such as the onset of symptoms, most recent episode, frequency and duration of episodes, and potential risk factors. The third part included four questions addressing the major signs and symptoms of BPPV, as outlined in the 2017 AAO-HNSF BPPV guidelines (10). These questions assessed whether vertigo (dizziness) was triggered by changes in head position (e.g., lying down, turning over in a supine position, or bending down), whether the duration of dizziness was 5–60 s, whether nausea was experienced, and whether blurry vision occurred.

### DHI

The severity and handicap associated with dizziness were evaluated before and after treatment using the DHI, developed by Jacobson and Newman in 1990 (11).

### Videonystagmography

Spontaneous nystagmus and positional nystagmus were recorded and analyzed before and after treatment using a videonystagmography system (Easy-eyes®; SLMED, Seoul, Republic of Korea). The video recording sequence included the following positions: sitting, lying down with the head centered, head rolled to the right side, head rolled to the left side, sitting, Dix–Hallpike maneuver on the right side, Dix–Hallpike maneuver on the left side, sitting, head hanging, sitting, and head bent (12, 13). Detailed results of the nystagmus analysis are presented in Supplementary Table S1.

### Repositioning maneuvers and follow-up

Patients with posterior canal (PC) BPPV were treated using the Epley maneuver followed by three consecutive Semont maneuvers (14, 15). Patients with horizontal canal (HC) BPPV underwent the barbecue roll maneuver followed by three consecutive Gufoni maneuvers (16, 17). Patients displaying anterior canal (AC) BPPV were treated with three consecutive Yacovino maneuvers (18). Patients were instructed to repeat the maneuvers at home three times daily for 7 days. Treatment outcomes were evaluated at 7, 14, and 28 days. If nystagmus persisted during a follow-up visit, treatment was continued, and the patient was re-evaluated at the subsequent follow-up.

### Bias

To minimize potential measurement bias, all patients were evaluated using standardized diagnostic criteria, evaluation tools (VNG, DHI), and treatment protocols. However, as this study was conducted at a single secondary referral center, selection bias may exist, and the findings may not be fully generalizable to primary care settings in Mongolia.

### Study size

The study size was determined by the number of eligible patients who presented to the clinic during the two-year study period and consented to participate. No *a priori* sample size calculation was performed as the primary objective was descriptive, aiming to assess the current status of medical services for BPPV.

### Cost analysis

Costs were estimated based on 2024 pricing to reflect current economic burdens, although clinical data were collected from 2019 to 2021.

### Statistical analysis

Patients with incomplete data were handled on an analysis-by-analysis basis; cases with missing values were excluded only from the specific analysis requiring that variable. Continuous variables are presented as mean ± SD and categorical variables as *n* (%). Between-group comparisons were performed using the chi-square test (or Fisher’s exact test when appropriate) for categorical variables and Student’s *t*-test for continuous variables; Welch’s *t*-test was used when variance assumptions were not met. Paired *t*-tests were used to compare pre- and post-treatment DHI scores.

For diagnostic delay, the interval from symptom onset to the BPPV diagnosis at the first specialist dizziness-clinic visit was calculated. For patients with pre-referral MRI, additional timing intervals were calculated using recorded dates: symptom onset to MRI acquisition, and MRI acquisition to the first specialist visit.

To identify factors associated with MRI usage, multivariate logistic regression analysis was performed. Clinical variables were entered into the model, and a stepwise approach was used to determine significant predictors. Results are presented as odds ratios (ORs) with 95% confidence intervals (CIs). A two-sided *p* < 0.05 was considered statistically significant. Statistical analyses were conducted using SPSS Statistics for Windows version 23.0 (IBM Corp., Armonk, NY, USA) and R version 4.5.2 (R Foundation for Statistical Computing, Vienna, Austria).

We prepared this manuscript following the STROBE (Strengthening the Reporting of Observational studies in Epidemiology) statement. The completed STROBE checklist is available as a Supplementary material.

## Results

### Baseline and clinical characteristics

The mean age of the patients was 50.0 ± 11.7 years (range, 22–82 years). Thirty-two patients (19.8%) were men, and 130 patients (80.2%) were women. The cause of BPPV was idiopathic in 115 patients (70.9%); the remaining cases were attributed to trauma (40 patients, 24.7%), prolonged bed rest (four patients, 2.5%), and otologic disease (three patients, 1.9%).

Among the 162 patients, 96 (59.3%) had PC BPPV, 44 (27.1%) had HC BPPV, 17 (10.5%) had AC BPPV, and five (3.1%) had multiple canal BPPV. HC BPPV was classified as geotropic in 21 (12.9%) patients and apogeotropic in 23 (14.2%) patients. The right ear was affected in 96 (59.3%) patients, the left ear in 60 (37.0%) patients, and both ears in two (1.2%) patients; the affected side was not determined in four (2.4%) patients (Table 1). Pearson’s chi-squared test revealed a higher incidence on the right side of the PC (*p* < 0.001) (Table 2).

**Table 1:** Descriptive statistics of total BPPV (*N* = 162).

Characteristics	*N* (%)	Missing *N* (%)
Affected canal		0 (0.0)
Posterior canal	96 (59.3)	
Horizontal canal (geotropic)	21 (12.9)	
Horizontal canal (apogeotropic)	23 (14.2)	
Anterior canal	17 (10.5)	
Multiple canal	5 (3.1)	
Affected side		4 (2.5)
Right	96 (59.3)	
Left	60 (37.0)	
Bilateral	2 (1.2)	
Initial vs. referral		0 (0.0)
Initial evaluation	60 (37)	
Referral from other hospital	102 (63)	
Referral diagnosis		0 (0.0)
BPPV	6 (3.7)	
No diagnosis	75 (46.3)	
Wrong diagnosis	21 (12.9)	

BPPV, Benign paroxysmal positional vertigo.

**Table 2:** Comparison of the lesion side between clinical types.

Affected canal (*N* = 162)	Posterior canal (*N* = 96)	Horizontal canal (*N* = 44)	Anterior canal (*N* = 17)	Multiple (*N* = 5)	*p*-value^c^
Right (*N* = 96)	61 (39.5)	22 (13.6)	10 (6.1)	3^a^	0.001
Left (*N* = 60)	35 (21.3)	22 (13.6)	3 (1.9)		
Bilateral (*N* = 2)		–	–	2^b^	–
Not determined (*n* = 4)	–	–	4 (2.4)		–

a Involved in both posterior canal and horizontal canal.

b Involved in bilateral posterior canal.

c Pearson’s *X*^2^ test.

As expected given the inclusion/diagnostic criteria based on the AAO-HNSF guideline framework, all patients responded “yes” to three key symptom questions: provocation by head position change, duration of 5–60 s, and nausea. In total, 142 patients (87.7%) also reported blurry vision.

### Diagnostic challenges: delays and misdiagnosis

The mean diagnostic delay (interval from symptom onset to BPPV diagnosis at the first specialist dizziness-clinic visit) was 16.7 ± 17.1 days. Of the 162 patients, 102 (62.5%) were initially evaluated at other hospitals before referral. BPPV was correctly diagnosed in only six patients (3.7%) before referral. The remaining patients were either undiagnosed (*n* = 75) or misdiagnosed (*n* = 21) with conditions such as transient ischemic attack or stroke (Table 1).

MRI was performed in 88 patients (54.3%) before their specialist visit (No MRI, *n* = 74). The interval from symptom onset to the diagnosis of BPPV was significantly longer in the MRI group (mean ± SD, 24.4 ± 19.5 days) than in the no-MRI group (7.5 ± 5.9 days). The mean difference was 16.8 days (95% CI, 12.5 to 21.1 days; *p* < 0.001, Welch’s *t*-test) (Table 3). The identified MRI centers are summarized in Supplementary Table S2.

**Table 3:** Association between MRI testing, diagnostic delay, and age of the patient.

Group	Underwent MRI (*n* = 88)	No MRI (*n* = 74)	*p*-value^a^
Duration (days)	24.4 ± 19.5	7.5 ± 5.9	**<0.001** ^**a**^
Age	51.2 ± 11.5	50.2 ± 12.2	**0.590** ^**a**^

MRI, magnetic resonance imaging. “Duration (days)” represents diagnostic delay from symptom onset to BPPV diagnosis.

a Welch’s two-sample *t*-test. Statistical significance was defined as *p* < 0.05.

To further characterize the timing of delays among patients who underwent MRI, we calculated the interval from symptom onset to MRI acquisition and from MRI acquisition to the first specialist visit. Among patients with MRI (*n* = 88), the mean (± SD) time from symptom onset to MRI was 11.3 ± 10.9 days, and the mean time from MRI to specialist visit was 13.1 ± 13.6 days (Table 4).

**Table 4:** Timing characteristics of pre-referral MRI utilization among BPPV patients who underwent MRI (*n* = 88).

Timing interval (days)	Mean ± SD	Median (IQR)	Range
Symptom onset → MRI	11.3 ± 10.9	8.0 (4.0–14.5)	1–64
MRI → Specialist visit (EMJJ)	13.1 ± 13.6	9.0 (3.8–17.0)	1–78
Symptom onset → Specialist visit (total delay)	24.4 ± 19.5	20.0 (12.0–30.0)	2–90

In addition, we reviewed the available imaging reports and medical records to describe MRI acquisition pathways (patient-initiated vs. clinician-associated) and facility type (private vs. public/unknown) in this cohort. These findings motivated an additional review of MRI acquisition pathways and facility type, as described below.

### MRI referral patterns and providers

Among the 88 patients who underwent pre-referral brain MRI, 68% (60/88) were documented as patient-initiated imaging (performed at the patient’s request or own decision according to available records). Among these patient-initiated cases (*n* = 60), 70% (42/60) underwent MRI in private hospitals, whereas the remaining 30% (18/60) were performed in public facilities or the facility name was not recorded. The referring specialty was not consistently documented; however, when identifiable, neurology was the most frequently recorded specialty (33 documented instances), followed by emergency physicians, general practitioners, and otolaryngologists.

### Treatment efficacy and quality of life improvement

**Figure 2:**
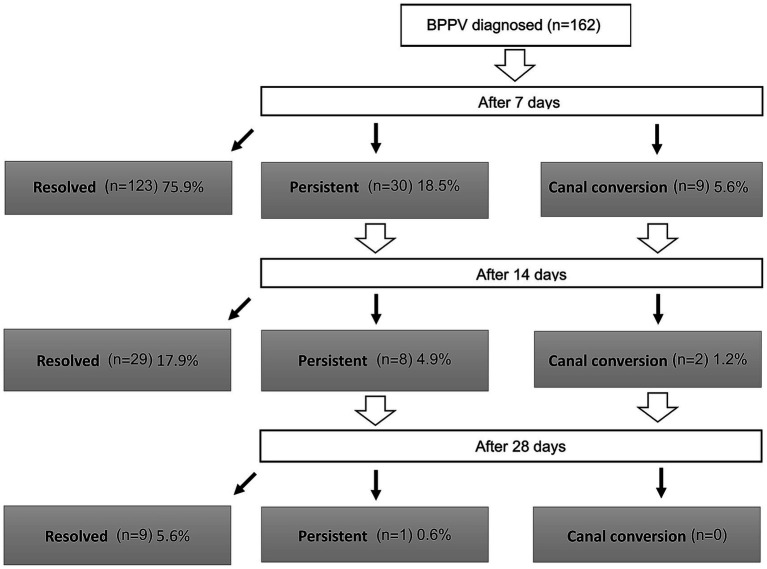
Flowchart of treatment outcomes for patients with benign paroxysmal positional vertigo (BPPV) (*n* = 162). This diagram illustrates the clinical course of patients at 7, 14, and 28 days after the initial canalith repositioning procedure. At each follow-up, patients were categorized as ‘Resolved’ (complete resolution of symptoms and nystagmus), ‘Persistent’ (continued symptoms and nystagmus), or ‘Canal conversion’ (development of nystagmus characteristic of a different semicircular canal). The number of patients in each category is shown, with the percentage calculated based on the initial total cohort of 162 patients.

Overall treatment resolution rates after 7, 14, and 28 days were 75.9% (95% CI 68.6–82.3), 93.8% (95% CI 88.9–97.0), and 99.4% (95% CI 96.6–100.0), respectively (Figure 2). When compared among clinical types, no significant differences in treatment success rates were observed (Table 5).

**Table 5:** Comparison of cumulative success rate of canalith repositioning maneuver.

Timepoint/Outcome	Posterior SCC (*n* = 101)^a^	Horizontal SCC (*n* = 44)	Anterior SCC (*n* = 17)
7 days later			
Resolved	78	34	11
Persistent	21	6	3
Canal conversion	2 (to AC 1, HC 1)	4 (Apo to Geo 2, Geo to Apo 1, Geo to PC 1)	3 (to PC)
Success rate of repositioning	77.2%	77.3%	64.7%
14 days later			
Resolved	17	7	5
Persistent	5	2	1
Canal conversion	1	1	–
Success rate of repositioning	73.9% (17/23)	70.0% (7/10)	83.3% (5/6)
28 days later			
Resolved	5	3	1
Persistent	1	–	–
Canal conversion	–	–	–
Success rate of repositioning	83.3% (5/6)	100%	100%

a For the cumulative success analysis by canal group, multiple-canal BPPV cases (*n* = 5) were included in the Posterior SCC group because all multiple-canal cases involved the posterior canal.

A significant improvement in DHI scores was observed following treatment (*p* < 0.001, paired *t*-test). The mean DHI score decreased from 39.93 ± 19.3 before treatment to 4.12 ± 4.6 after treatment (mean difference 35.8, 95% CI 32.9 to 38.7; *p* < 0.001). Before treatment, 52.46% of patients reported a moderate or severe handicap; after treatment, 99.4% reported no handicap (Table 6).

**Table 6:** Dizziness Handicap Inventory score before and after treatment.

Affected canal	Dizziness Handicap Inventory (mean ± SD)		*p*-value^a^
	Before treatment	After treatment	
Posterior canal	40.8 ± 19.6	3.9 ± 4.7	<0.001
Horizontal canal	38.2 ± 16.5	4.2 ± 4.3	<0.001
Anterior canal	39.3 ± 23.9	5.3 ± 5.6	<0.001
Overall	39.9 ± 19.3	4.1 ± 4.6	<0.001

a Paired *t*-test.

### Factors associated with MRI usage and diagnostic delay

Patients who underwent MRI scans experienced a significantly longer delay from symptom onset to diagnosis (24.4 ± 19.5 days) compared to those who did not (7.5 ± 5.9 days; *p* < 0.001). However, in the multivariate logistic regression analysis, no specific clinical symptoms (e.g., hearing loss, severe nausea, or type of vertigo) were found to be significant predictors of MRI usage (all *p* > 0.05). Interestingly, the variable ‘no change in hearing’ was retained in the final stepwise model with an Odds Ratio of 2.18 (95% CI 0.77–7.14, *p* = 0.163), suggesting a paradoxical trend where patients without auditory symptoms were potentially more likely to undergo neuroimaging, although this did not reach statistical significance.

Clinical symptoms mimicking central pathology, such as hearing loss or continuous vomiting, were observed in less than 3% of patients. While all patients with these specific red flags underwent MRI (100%), they were excluded from the multivariate model due to their rarity causing complete separation. In the final stepwise logistic regression model excluding these sparse variables, no remaining clinical symptoms (e.g., visual blurring, tinnitus) significantly predicted MRI usage. This suggests that MRI utilization may be influenced by factors beyond the recorded clinical symptoms, rather than by specific “red flag” symptoms alone.

### Economic impact and financial toxicity

Based on 2024 healthcare cost metrics in Ulaanbaatar, we estimated the financial burden of potentially low-yield imaging. The average cost of a brain MRI in a mid-tier private hospital was estimated at 475,000 MNT (~$140 USD), whereas the specialist consultation fee was approximately 50,000 MNT (~$15 USD), yielding a cost ratio of 9.5.

For a median income earner in Mongolia, one MRI scan represents 21.3% of their monthly income. For minimum wage workers, the financial toxicity reaches catastrophic levels, with a single scan consuming 72.0% of their monthly earnings. In our cohort, the 88 potentially low-yield MRIs resulted in a direct waste of approximately 41.8 million MNT (~$12,300 USD), excluding indirect productivity losses. The aggregated wasted cost of 41.8 million MNT observed in this single-center cohort alone could have covered the cost of establishing basic vestibular screening kits (e.g., Frenzel goggles) for dozens of primary care clinics.

## Discussion

This study reviewed the clinical characteristics and treatment outcomes of BPPV in Mongolia, reflecting the current state of medical services for this common condition. Our study highlights a “diagnostic paradox” typical of healthcare systems in transition. While Mongolia’s 16-day diagnostic delay is an improvement over the chronic delays reported in China (>70 months) (1) and Pakistan (19 months) (2), the reliance on high-cost imaging remains a critical inefficiency. Our MRI utilization rate of 54.3% mirrors the rates seen in tertiary centers in China and India, suggesting that while access to care has improved, the quality of triage has not kept pace with the availability of technology. Despite these diagnostic delays and initial challenges, treatment success was high, with 99.4% of patients recovered by day 28, accompanied by significant improvement in Dizziness Handicap Inventory (DHI) scores after treatment.

In terms of demographic and canal characteristics, our findings on sex distribution (a male-to-female ratio of 1:4) and a mean patient age of 50 years are generally consistent with the published literature (19). The predominance of right-sided involvement in posterior canal (PC) BPPV is also a common finding, often attributed to sleeping position (20). However, the proportion of anterior canal (AC) BPPV in our cohort (10.5%) was higher than the 3% reported in some comparable studies (21). This may reflect differences in diagnostic practice and may be partially influenced by diagnostic overlap with uncommon variants such as apogeotropic posterior canal BPPV (short-arm variant), which can mimic anterior canal involvement by presenting with downbeat positional nystagmus.

The extended diagnostic delay, as highlighted above, is likely due to limited awareness and confidence in vestibular examination among primary care physicians (PCPs), a lack of specialized facilities outside the capital city, and geographic barriers. This aligns with global observations that PCPs often lack the training to confidently diagnose BPPV at the initial point of care (22, 23). Notably, our multivariate analysis failed to identify any specific clinical symptoms that predicted MRI usage.

Notably, our additional pathway review suggested that MRI utilization was frequently driven by patient-initiated demand and private-sector access: 68% (60/88) of MRIs were documented as patient-initiated; among these patient-initiated cases, 70% (42/60) were performed in private facilities. In the local context, MRI access in the public sector may involve substantial waiting times (reported to be several months), whereas private centers can provide more immediate imaging. This system-level disparity, combined with health anxiety regarding stroke, may contribute to early imaging before a structured bedside vestibular evaluation. Importantly, we do not discourage neuroimaging when central red flags are present; rather, our findings highlight that, among patients ultimately diagnosed with BPPV, an imaging-first pathway may increase diagnostic delay and financial burden without improving diagnostic yield.

Importantly, our message is not to discourage neuroimaging when central red flags are present. Rather, our timing analysis suggests that, among patients ultimately diagnosed with BPPV, imaging-first pathways may contribute to delays at multiple points in the diagnostic journey: from symptom onset to imaging acquisition and from imaging acquisition to specialist evaluation (Table 4). In resource-limited settings, the relative financial impact of MRI is substantial, underscoring the importance of educational interventions and structured bedside triage to reduce unwarranted investigations (24, 25).

These findings have important implications for clinicians and policymakers in Mongolia and other developing countries facing similar healthcare challenges. The significant diagnostic delays and high rates of misdiagnosis highlight the urgent need for targeted educational programs for general practitioners (GPs) and the public to improve early recognition and referral of BPPV (23, 26). Global evidence strongly supports that even brief, focused training interventions can markedly improve PCPs’ diagnostic accuracy and confidence in BPPV management (8). Implementing simple, cost-effective canalith repositioning procedures at an earlier stage has been shown to substantially reduce unnecessary diagnostic tests and specialist referrals (26). By empowering PCPs to manage BPPV at the primary care level, healthcare systems can improve efficiency and patient quality of life, particularly in developing countries with limited access to specialists (27).

The economic implications of this practice are profound. Our cost analysis demonstrates that a single MRI scan consumes 72.0% of a minimum wage worker’s monthly income, qualifying as catastrophic health expenditure. In resource-limited settings, the opportunity cost is high; the resources wasted on the 88 negative MRIs in this study could have funded nearly 850 specialist consultations. Transitioning from a “technology-first” to a “technique-first” approach by training primary care providers in vestibular maneuvers represents a high-yield intervention for reducing both diagnostic delay and economic waste. Although the clinical data were collected retrospectively (2019–2021), the cost analysis was performed using 2024 pricing models to calculate the current financial toxicity and provide relevant policy implications for the present healthcare system.

This study has several strengths and limitations. To the best of our knowledge, this is the first study to analyze the clinical characteristics and treatment outcomes of BPPV in Mongolia using standardized diagnostic criteria and videonystagmography. By analyzing a relatively large cohort from a single secondary referral center, we provided a consistent dataset that highlights the “diagnostic paradox” in a developing country setting. However, several limitations should be considered. First, as a single-center study conducted at a secondary referral hospital, the findings may not be fully generalizable to primary care settings across the entire country. Second, the retrospective design limits our ability to establish causal relationships. Third, inherent selection bias exists because our cohort consisted only of patients definitively diagnosed with BPPV. Patients with central pathologies (e.g., stroke) identified by MRI at other facilities would likely have been referred to neurology departments. Therefore, while our data imply a near-zero diagnostic yield for MRI in this specific cohort, it highlights the failure of initial triage rather than the diagnostic inutility of MRI for all undifferentiated dizziness. Finally, specific BPPV risk factors, such as vitamin D deficiency and osteoporosis, were not systematically assessed (28, 29).

Future research should explore the prevalence of BPPV risk factors such as vitamin D deficiency and osteoporosis in the Mongolian population and assess their impact on recurrence or severity (28). Further analysis could also examine the relationship between diagnostic delay and initial DHI scores or long-term quality of life outcomes. Finally, longitudinal studies are needed to evaluate the effectiveness of awareness campaigns and training programs for primary care providers, building on international experiences that have demonstrated measurable improvements in BPPV care.

## Data Availability

The raw data supporting the conclusions of this article will be made available by the authors, without undue reservation.

## In memoriam

We acknowledge the late Professor Seung Ha Oh for his mentorship and guidance that contributed to the development of this study. This work is dedicated to his memory.

## References

[1] Wang H Yu D Song N Su K Yin S. Delayed diagnosis and treatment of benign paroxysmal positional vertigo associated with current practice. Eur Arch Otorrinolaringol. (2014) 271:261–4. doi: 10.1007/s00405-012-2333-8, 23455578

[2] Arshad M Abbas S Qureshi IA. Delay in diagnosis and treatment of benign paroxysmal positional vertigo in current practice. J Ayub Med Coll Abbottabad. (2013) 25:93–5. 25098065

[3] Grill E Strupp M Muller M Jahn K. Health services utilization of patients with vertigo in primary care: a retrospective cohort study. J Neurol. (2014) 261:1492–8. doi: 10.1007/s00415-014-7367-y, 24817192

[4] Moon SY Kim JS Kim BK Kim JI Lee H Son SI . Clinical characteristics of benign paroxysmal positional vertigo in Korea: a multicenter study. J Korean Med Sci. (2006) 21:539–43. doi: 10.3346/jkms.2006.21.3.539, 16778402 PMC2729964

[5] Cengiz DU Demir I Demirel S Can Colak S Emekci T Bayindir T. Investigation of the relationship between BPPV with anxiety, sleep quality and falls. Turk Arch Otorhinolaryngol. (2022) 60:199–205. doi: 10.4274/tao.2022.2022-8-6, 37456598 PMC10339271

[6] Yeo BSY Toh EMS Lim NE Lee RS Ho RCM Tam WWS . Association of benign paroxysmal positional vertigo with depression and anxiety-a systematic review and meta-analysis. Laryngoscope. (2024) 134:526–34. doi: 10.1002/lary.30957, 37560919

[7] Lopez-Escamez JA Gamiz MJ Fernandez-Perez A Gomez-Finana M. Long-term outcome and health-related quality of life in benign paroxysmal positional vertigo. Eur Arch Otorrinolaringol. (2005) 262:507–11. doi: 10.1007/s00405-004-0841-x 15942805

[8] Ballve JL Carrillo-Munoz R Rando-Matos Y Villar I Cunillera O Almeda J . Effectiveness of the Epley manoeuvre in posterior canal benign paroxysmal positional vertigo: a randomised clinical trial in primary care. Br J Gen Pract. (2019) 69:e52–60. doi: 10.3399/bjgp18X700253, 30510098 PMC6301349

[9] Gamiz MJ Lopez-Escamez JA. Health-related quality of life in patients over sixty years old with benign paroxysmal positional vertigo. Gerontology. (2004) 50:82–6. doi: 10.1159/000075558, 14963374

[10] Bhattacharyya N Gubbels SP Schwartz SR Edlow JA El-Kashlan H Fife T . Clinical practice guideline: benign paroxysmal positional vertigo (update). Otolaryngol Head Neck Surg. (2017) 156:S1–S47. doi: 10.1177/0194599816689667, 28248609

[11] Jacobson GP Newman CW. The development of the Dizziness Handicap Inventory. Arch Otolaryngol Head Neck Surg. (1990) 116:424–7. doi: 10.1001/archotol.1990.01870040046011 2317323

[12] Fife TD Iverson DJ Lempert T Furman JM Baloh RW Tusa RJ . Practice parameter: therapies for benign paroxysmal positional vertigo (an evidence-based review): report of the quality standards subcommittee of the American Academy of Neurology. Neurology. (2008) 70:2067–74. doi: 10.1212/01.wnl.0000313378.77444.ac 18505980

[13] Furman JM Cass SP. Benign paroxysmal positional vertigo. N Engl J Med. (1999) 341:1590–6. doi: 10.1056/NEJM199911183412107 10564690

[14] Epley JM. The canalith repositioning procedure: for treatment of benign paroxysmal positional vertigo. Otolaryngol Head Neck Surg. (1992) 107:399–404. doi: 10.1177/019459989210700310 1408225

[15] Semont A Freyss G Vitte E. Curing the BPPV with a liberatory maneuver. Adv Otorhinolaryngol. (1988) 42:290–3. doi: 10.1159/000416126, 3213745

[16] Lempert T Tiel-Wilck K. A positional maneuver for treatment of horizontal-canal benign positional vertigo. Laryngoscope. (1996) 106:476–8. doi: 10.1097/00005537-199604000-00015 8614224

[17] De la Meilleure G Dehaene I Depondt M Damman W Crevits L Vanhooren G. Benign paroxysmal positional vertigo of the horizontal canal. J Neurol Neurosurg Psychiatry. (1996) 60:68–71. doi: 10.1136/jnnp.60.1.68, 8558155 PMC486192

[18] Yacovino DA Hain TC Gualtieri F. New therapeutic maneuver for anterior canal benign paroxysmal positional vertigo. J Neurol. (2009) 256:1851–5. doi: 10.1007/s00415-009-5208-1, 19536580

[19] Teggi R Guidetti R Gatti O Guidetti G. Recurrence of benign paroxysmal positional vertigo: experience in 3042 patients. Acta Otorhinolaryngol Ital. (2021) 41:461–6. doi: 10.14639/0392-100X-N1233, 34734582 PMC8569667

[20] Yousovich R Duvdevani SI Lipschitz N Wolf M Migirov L Yakirevitch A. Correlation between the sleep-position habits and the affected posterior semicircular canal in patients with benign paroxysmal positional vertigo. Isr Med Assoc J. (2019) 21:716–8. 31713357

[21] Anagnostou E Kouzi I Spengos K. Diagnosis and treatment of anterior-canal benign paroxysmal positional vertigo: a systematic review. J Clin Neurol. (2015) 11:262–7. doi: 10.3988/jcn.2015.11.3.262, 26022461 PMC4507381

[22] Ulyte A Valancius D Masiliunas R Paskoniene A Lesinskas E Kaski D . Diagnosis and treatment choices of suspected benign paroxysmal positional vertigo: current approach of general practitioners, neurologists, and ENT physicians. Eur Arch Otorrinolaringol. (2019) 276:985–91. doi: 10.1007/s00405-019-05313-y, 30694376

[23] Alshehri S Al Ahmree AO Qobty A Musleh A Alahmari KA. Variations in the diagnosis and management of benign paroxysmal positional vertigo among physician specialties in Saudi Arabia: influence of clinical experience and case exposure. Healthcare (Basel). (2025) 13:1887. doi: 10.3390/healthcare13151887, 40805918 PMC12345922

[24] Newman-Toker DE Camargo CA Jr Hsieh YH Pelletier AJ Edlow JA. Disconnect between charted vestibular diagnoses and emergency department management decisions: a cross-sectional analysis from a nationally representative sample. Acad Emerg Med. (2009) 16:970–7. doi: 10.1111/j.1553-2712.2009.00523.x 19799573

[25] Silva AL Marinho MR Gouveia FM Silva JG Ferreira Ade S Cal R. Benign paroxysmal positional vertigo: comparison of two recent international guidelines. Braz J Otorhinolaryngol. (2011) 77:191–200. doi: 10.1590/s1808-86942011000200009, 21537621 PMC9450775

[26] Meurer WJ Beck KE Rowell B Brown D Tsodikov A Fagerlin A . Implementation of evidence-based practice for benign paroxysmal positional vertigo: DIZZTINCT—a study protocol for an exploratory stepped-wedge randomized trial. Trials. (2018) 19:697. doi: 10.1186/s13063-018-3099-0, 30577834 PMC6303863

[27] Oghalai JS Manolidis S Barth JL Stewart MG Jenkins HA. Unrecognized benign paroxysmal positional vertigo in elderly patients. Otolaryngol Head Neck Surg. (2000) 122:630–4. doi: 10.1016/S0194-5998(00)70187-2, 10793337

[28] Byun H Chung JH Lee SH Park CW Kim EM Kim I. Increased risk of benign paroxysmal positional vertigo in osteoporosis: a nationwide population-based cohort study. Sci Rep. (2019) 9:3469. doi: 10.1038/s41598-019-39830-x, 30837524 PMC6401187

[29] Chen J Zhao W Yue X Zhang P. Risk factors for the occurrence of benign paroxysmal positional vertigo: a systematic review and meta-analysis. Front Neurol. (2020) 11:506. doi: 10.3389/fneur.2020.00506, 32655479 PMC7324663

