# Supplementary material for: Unnecessary magnetic resonance imaging is associated with diagnostic delay and financial burden in benign paroxysmal positional vertigo: a retrospective study in Mongolia
Source: Front Neurol. 2026 Feb 13;17:1777303. doi: 10.3389/fneur.2026.1777303 (PMC12945807; doi:10.3389/fneur.2026.1777303)
Supplement: Supplementary file 2 [file Table_1.docx]

**Supplementary Table S1.** Nystagmus analysis assuming BPPV in right ear

| BPPV (N=157) | Nystagmus | Lie down  n,(%) | Head roll Right  n,(%) | Head roll Left n,(%) | Dix-Hallpike Right n,(%) | Dix-Hallpike Left n,(%) | Head Bend n,(%) | Head Hang n,(%) |
| --- | --- | --- | --- | --- | --- | --- | --- | --- |
| Posterior canal (N=96) | UB | 4 (4.2) | - | - | 2 (2.1) | - | 1 (1) | 1 (1) |
|  | UB, CW | 7 (7.3) | 25 (26) | 1 (1) | 77 (80.2) | - | - | 5 (5.2) |
|  | UB, CCW | - | - | - | - | - | 1 (1) | - |
|  | UB, RB | - | - | - | 1 (1) | - | - | - |
|  | DB | - | - | - | - | - | 9 (9.4) | 1 (1) |
|  | DB, CW | - | - | - | 1 (1) | - | 6 (6.2) | - |
|  | DB, CCW | - | 1 (1) | - | - | - | 5 (5.2) | - |
|  | RB | - | 1 (1) | 3 (3.1) | - | - | - | - |
| Horizontal canal Geotropic  (N=21) | RB | - | 18 (85.7) | - | 4 (19) | 4 (19) | 6 (28.5) | - |
|  | LB | 15 (71.4) | - | 19 (90.4) | - | - | - | 2 (9.5) |
| Horizontal canal Apo-geotropic  (N=23) | RB | 15 (65.2) | - | 12 (52) | - | 7 (30) | - | - |
|  | LB | - | 15 (65.2) | - | 4 (17) | - | 3 (13) | - |
| Anterior canal  (N=17) | DB | 2 (11.7) | 2 (11.7) | 2 (11.7) | 9 (52.9) | 6 (35.2) | 2 (11.7) | 9 (52.9) |
|  | DB, CW | 1 (5.8) | 10 (58.8) | - | 13 (76.4) | 3 (17.6) | - | 8 (5.8) |

Note: UB-Upbeat; DB-Downbeat; RB-Right beat; LB-Left beat; CW-Clockwise; CCW-Counter clockwise; BPPV- Benign paroxysmal positional vertigo;

**Supplementary Table S2.** MRI centers identified from imaging reports (n=88)

| Hospital Name | Type |
| --- | --- |
| Medtrauma Hospital | Private |
| UB Songdo Hospital | Private |
| Luxmed Hospital | Private |
| Mungunguur Hospital | Private |
| Intermed Hospital | Private |
| Grandmed Hospital | Private |
| Others / Unspecified* | Public / State Hospitals |

Note: Specific facility names were not consistently recorded on MRI report forms issued by public institutions during the study period (2019–2021). Therefore, reports lacking a specific private hospital identifier were categorized as originating from public tertiary hospitals.
